# A Direct Electric Field-Aided Biomimetic Mineralization System for Inducing the Remineralization of Dentin Collagen Matrix

**DOI:** 10.3390/ma8115433

**Published:** 2015-11-20

**Authors:** Xiao-Ting Wu, May Lei Mei, Quan-Li Li, Chris Ying Cao, Jia-Long Chen, Rong Xia, Zhi-Hong Zhang, Chun Hung Chu

**Affiliations:** 1 College & Hospital of Stomatology, Anhui Medical University, Key Laboratory of Oral Diseases Research of Anhui Province, Hefei 230032, China; yimi892680094@163.com (X.-T.W.); jialong_dt@126.com (J.-L.C.); 2 Faculty of Dentistry, The University of Hong Kong, Hong Kong 999077, China; mei1123@hku.hk (M.L.M.); caoying0713@gmail.com (C.Y.C.); chchu@hku.hk (C.H.C.); 3 Department of Stomatology, The Second Hospital Affiliated to Anhui Medical University, Hefei 230601, China; xiarongqh@aliyun.com; 4 Department of Stomatology, The Hospital of Anhui Province, Hefei 230001, China; zzhzqr@126.com

**Keywords:** dentin, demineralization, collagen matrix, biomimetic mineralization, electric field

## Abstract

This *in vitro* study aimed to accelerate the remineralization of a completely demineralized dentine collagen block in order to regenerate the dentinal microstructure of calcified collagen fibrils by a novel electric field-aided biomimetic mineralization system in the absence of non-collagenous proteins. Completely demineralized human dentine slices were prepared using ethylene diamine tetraacetic acid (EDTA) and treated with guanidine hydrochloride to extract the bound non-collagenous proteins. The completely demineralized dentine collagen blocks were then remineralized in a calcium chloride agarose hydrogel and a sodium hydrogen phosphate and fluoride agarose hydrogel. This process was accelerated by subjecting the hydrogels to electrophoresis at 20 mA for 4 and 12 h. X-ray diffraction (XRD), scanning electron microscopy (SEM), energy dispersive X-ray spectroscopy (EDX), and transmission electron microscopy (TEM) were used to evaluate the resultant calcification of the dentin collagen matrix. SEM indicated that mineral particles were precipitated on the intertubular dentin collagen matrix; these densely packed crystals mimicked the structure of the original mineralized dentin. However, the dentinal tubules were not occluded by the mineral crystals. XRD and EDX both confirmed that the deposited crystals were fluorinated hydroxyapatite. TEM revealed the existence of intrafibrillar and interfibrillar mineralization of the collagen fibrils. A novel electric field-aided biomimetic mineralization system was successfully developed to remineralize a completely demineralized dentine collagen matrix in the absence of non-collagenous proteins. This study developed an accelerated biomimetic mineralization system which can be a potential protocol for the biomineralization of dentinal defects.

## 1. Introduction

Dentin is the main component of teeth. Structurally, the basic microstructure of dentin is composed of a calcified collagen matrix, which is made up of inorganic hydroxyapatite (HA) based minerals (approximately 70% by weight), organic matrix (20%), and water (10%). The main organic substance of dentin is type I collagen, and a small amount of non-collagenous proteins (NCPs) is also included. Collagen self assembles into fibrils to form collagen matrix scaffolds, which act as templates for mineral formation. NCPs are believed to play a crucial role in the regulation of mineralization [1]. Caries and acid erosion often cause dentin demineralization, which results in the exposure of the collagen matrix and leads to further degradation. At present, it is very important to duplicate the calcified collagen matrix in order to enable us to induce the self-healing of a dentinal defect. The remineralization of dentin collagen fibrils is far more difficult and less effective than the remineralization of enamel. Fan *et al.* [2] reported that under the same remineralizing condition, remineralization occurred on the surface of acid-etched enamel but not on the surface of acid-etched dentin. This difference could be attributed to the presence of fewer residual mineral crystals and the exposure of organic matrix (mainly type I collagen) on the acid-etched dentin surface. Thus, the classical ion-based crystallization concept may not be applicable for remineralizing completely demineralized dentin [3]. In recent years, several approaches, such as those using supersaturated calcium phosphate solution, bioactive glass particles [4], polydopamine [5], calcium phosphate-loaded gel [6], and casein phosphopeptide-amorphous calcium phosphate (CPP-ACP) [7], that could only induce HA to deposit on the dentin surface, which were not representative of the real remineralization of dentin collagen fibrils. However, the biomimicry of NCP-using substances such as polyacrylic acid (PAA), polyvinylphosphonic acid (PVPA) [8], sodium trimetaphosphate (STMP) [9], oligopeptides inspired by NCPs [10], polymer-induced liquid precursors that use poly-l-aspartate sodium salt (polyAsp) to stabilize a precursor phase of amorphous calcium phosphate (ACP) [11], and poly(amidoamine) dendrimers [12], have been shown to remineralize the dentin collagen matrix by duplicating the dentin microstructure within calcified collagen fibrils. However, the rate of mineralization is very slow in the aforementioned methods, which limits their clinical application. Moreover, only a thin layer of the dentin surface (<10 μm) could be demineralized in the aforementioned studies. Remnant apatite crystallite seeds under the demineralized dentin surface can act as centers for heterogeneous nucleation [13]. The remineralization of a partially demineralized collagen matrix is thermodynamically more favorable than that of a completely demineralized collagen matrix with homogeneous nucleation. Therefore, a model of surface-demineralized dentin may be easier to remineralize than a completely demineralized one. To date, no reports have examined the remineralization of a completely demineralized dentin collagen matrix block. Moreover, an understanding of the mechanism behind dentin collagen mineralization might be achieved by using a completely demineralized dentin collagen matrix block. The advantages of using a completely demineralized dentin collagen matrix block include the elimination of the ambiguity that arises when trying to discern the remineralized apatite crystallites from the remnant apatite seed crystallites and the ability to ascertain whether the remineralization of collagen matrix can occur in the absence of NCPs.

In a previous study, we developed a technique to remineralize dentin collagen fibrils in partially demineralized dentin slices using the aid of electrophoresis [14]. We successfully regenerated the dentin microstructure of a calcified collagen matrix in the absence of both NCPs and their analogs and greatly accelerated the speed of mineralization. We hypothesized that the application of an electric field might play an important role in promoting the mineralization process. In the present study, we hypothesized that this powerful technique could also remineralize a completely demineralized dentin collagen matrix block and aimed to both regenerate the tooth microstructure and to shorten the mineralization time.

## 2. Results

**Figure 1:**
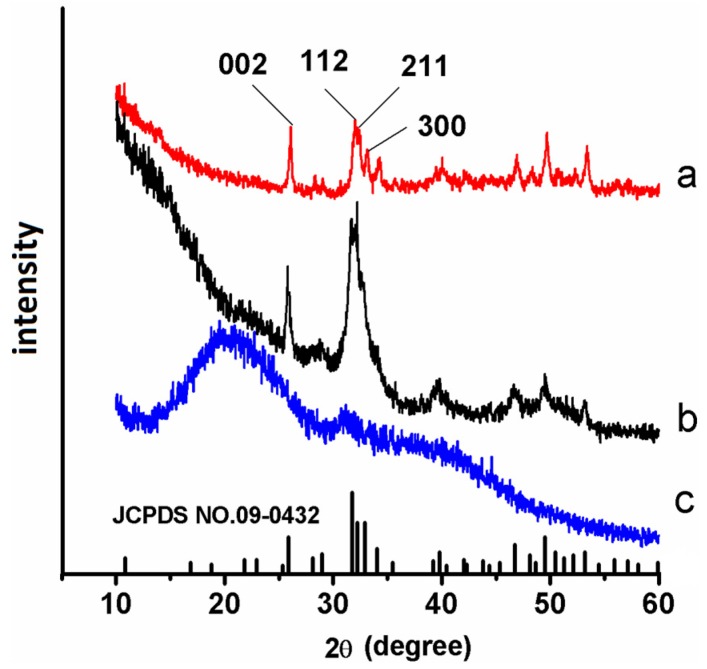
X-ray diffraction (XRD) spectra of the precipitates that were formed on dentin after six cycles of electric field-aided mineralization (**a**, red line), XRD spectra of sound dentin (**b**, black line) and completely demineralized dentin (**c**, blue line).

The X-ray diffraction (XRD) spectra confirmed that the precipitates that formed on the remineralized dentin slices were fluorinated HA. Spectrum (a) in Figure 1 shows the XRD pattern of the crystals that grew on the completely demineralized dentin surface after remineralization for six cycles in the electric field-aided biomimetic mineralization system. The diffraction peaks (002) at 2θ = 25.8, (211) at 2θ = 31.9, (112) at 2θ = 32.4, and (300) at 2θ = 33.3 corresponded well with the expected peaks of HA, respectively (JCPDS NO. 09-0432) [2]. The ratio of the diffraction intensity of the c-axis (002) reflection to the diffraction intensity of the a-axis (300) reflection for each experimental slice was considerably greater than that of the sound dentin slice (spectrum b). This result suggested that the HA precipitates were oriented along the c-axis.

**Figure 2:**
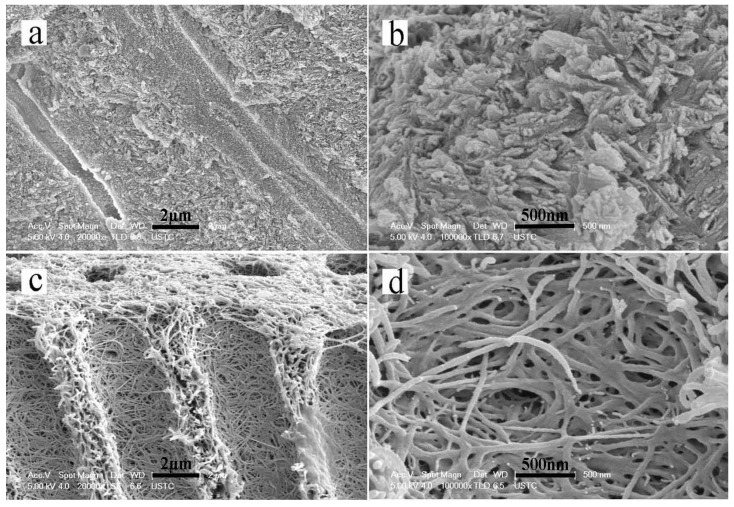
Scanning electron microscopy (SEM) micrographs showing a cross-sectional view of sound dentin and completely demineralized dentin. (**a**,**b**) The structure of the tubules and well mineralized intertubular dentin of sound dentin. (**c**,**d**) The structure of completely demineralized dentin. All of the minerals on the surfaces and interiors of the dentin slices were dissolved and had disappeared.

The SEM results showed that the sound dentin consisted of well-mineralized peritubular and intertubular dentin in addition to dentinal tubules (Figure 2a,b). The dentin had been completely demineralized by EDTA after 20 days. All of the minerals on the surfaces and interiors of the dentin slices had dissolved and disappeared. The dentin collagen matrix was exposed and exhibited a soft property. Additionally, the dentinal tubules became obvious and had enlarged diameters (Figure 2c,d).

**Figure 3:**
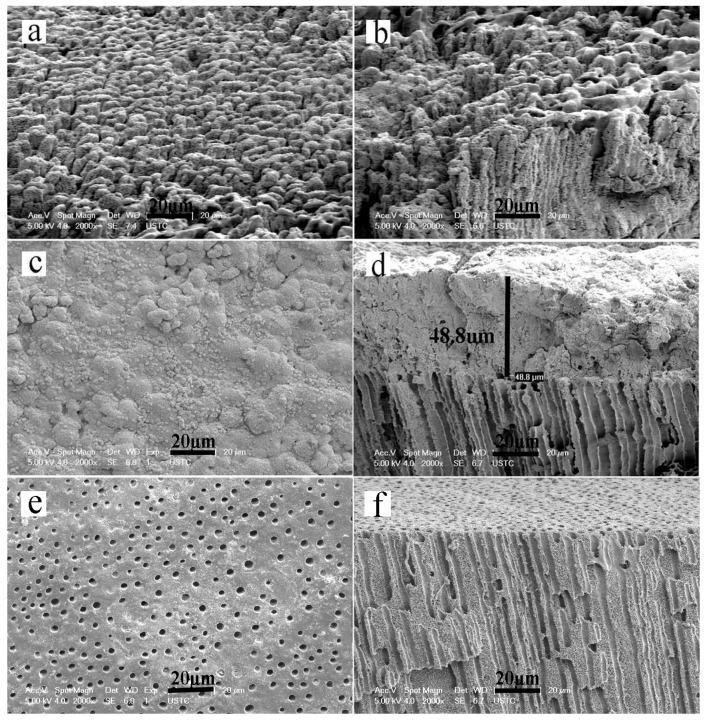
SEM micrographs of remineralized dentin slices. Panels a, c and e are surface images. Panels b, d, and f are cross-sectional views of the samples shown in panels a, c, and e, respectively. (**a**,**b**) The minerals deposited on the dentin surface after two cycles; (**c**,**d**) The minerals deposited on the dentin surface after six cycles of remineralization; (**e**,**f**) The minerals deposited in the control group that did not include the aid of an electric field after six cycles of remineralization.

The minerals that were deposited on the dentin surface formed a honeycomb-like morphology, and the collagen matrix became stiff after two mineralization cycles (4 h) (Figure 3a,b). After six mineralization cycles (12 h), the HA crystals packed together densely to form a mineral layer on the dentin surface that completely covered the underlying dentin collagen matrix (Figure 3c,d). However, in the control group that was not exposed to an electric field, only a surface deposition of scattered apatite clusters were found on the demineralized collagen after six mineralization cycles (12 h). There were no detectable minerals in the interior collagen (Figure 3e,f).

**Figure 4:**
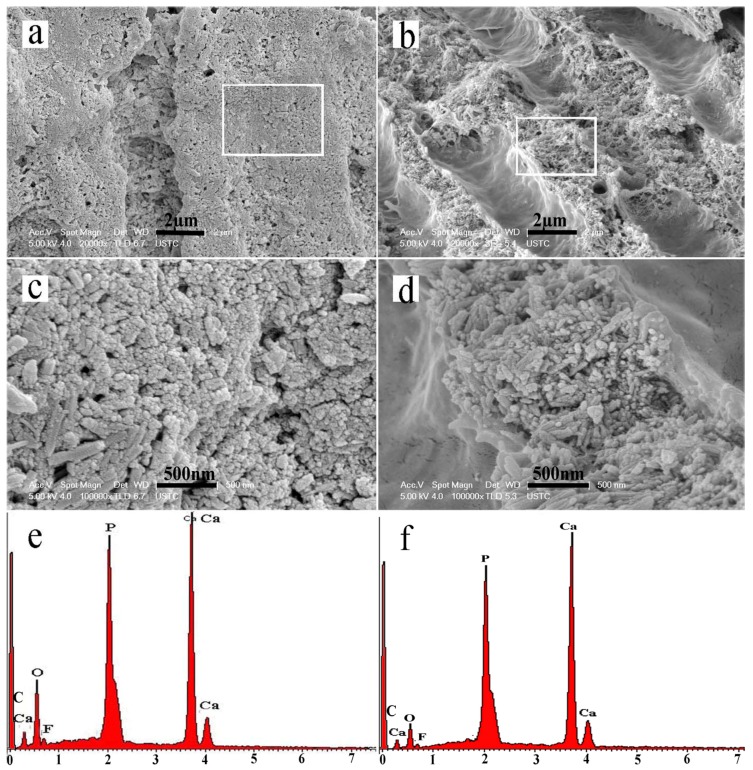
SEM micrographs of well-remineralized intertubular dentin after six remineralization cycles. (**a**) The intertubular dentin near the dentin surface; (**b**) The intertubular dentin in the interior of the dentin slice; (**c**,**d**) Magnified micrograph of the region denoted by a rectangle in (**a**) and (**b**), respectively; (**e**,**f**) Dispersive X-ray spectroscopy (EDX) of the remineralized intertubular dentin in (**b**) and (**d**).

After remineralization for six minerialization cycles (12 h), numerous HA crystals precipitated on the intertubular dentin near the dentin surface. The dentin collagen fibrils were well mineralized, which made the dentinal collagen almost invisible. Additionally, numerous crystals had also precipitated on the walls of the dentinal tubules (Figure 4a,c). In the interior of the dentin, the intertubular dentin was also well mineralized. The well-remineralized intertubular dentin (Figure 4c,d) seemed extremely similar to the appearance of mineralized sound dentin of calcified collagen matrix (Figure 2b). However, there was nearly no deposition of crystal on the walls of the dentinal tubules and therefore the opened and enlarged tubules were hardly occluded (Figure 4b,d). Dispersive X-ray spectroscopy (EDX) (Figure 4e,f) showed that calcium, oxygen, fluorine, and phosphorus were the main elements comprising the intertubular dentin. The Ca/P ratio of the regenerated crystals was 1.70, which revealed that the crystals were similar to the HA (Ca/P 1.67) of sound tooth dentin.

**Figure 5:**
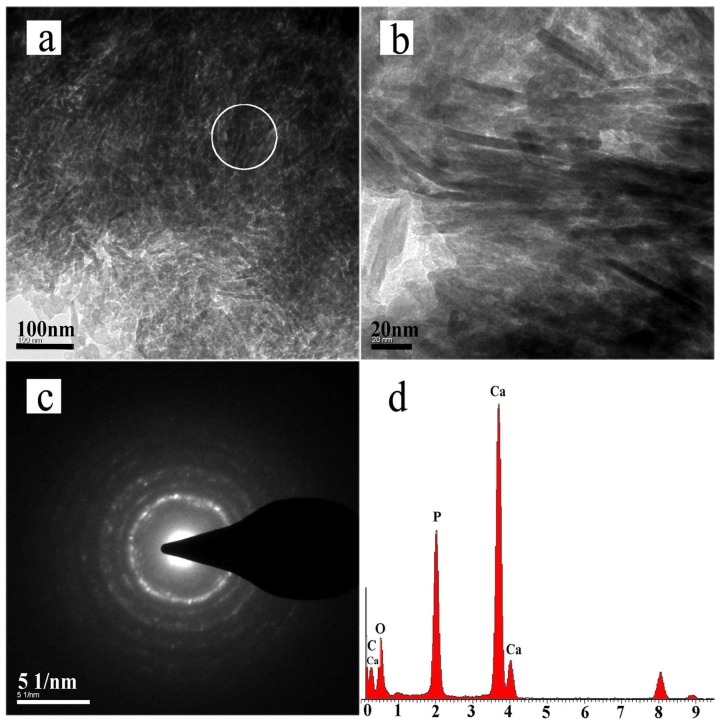
Transmission electron microscopy (TEM) micrographs of dentin remineralized after six remineralization cycles. (**a**) TEM bright-field micrographs of unstained remineralized dentin collagen fibrils after six remineralization cycles; (**b**) Magnified view of panel a; (**c**) Selected area electron diffraction (SAED) pattern of remineralized dentin collagen fibrils; (**d**) EDX spectra of the remineralized fibrils.

Transmission electron microscopy (TEM) bright-field micrographs enabled the visualization of the remineralized dentin collagen fibrils, which were examined after six cycles of remineralization (Figure 5a,b). The fibrils showed a dark contrast, discrete electron dense islands, and areas where the collagen fibrils were difficult to distinguish. The samples were not stained with phosphotungstic acid or any other electron-dense substance and therefore these observations suggested that the collagen fibrils were highly mineralized. Both the intrafibrillar mineralization of collagen fibrils and the interfibrillar mineralization of the matrix adjacent to the fibrils could be observed in the electron-dense images. The selected area electron diffraction (SAED) pattern of the precipitates revealed discrete string-like patterns that were characteristic of HA (Figure 5c). The EDX spectrum showed that calcium, phosphate, and oxygen were present in the remineralized collagen fibrils (Figure 5d).

## 3. Discussion

To retain an intact dentinal collagen matrix, EDTA was used to completely demineralize the dentin slices. EDTA differs from the aggressive agents that have previously been used, such as formic acid/sodium formate, phosphoric acid and hydrochloric acid. EDTA is generally accepted as the most effective chelating agent that is capable of dissolving the mineral phase in bone by complexation while keeping the collagen superstructure essentially intact, which holds true even when soaking the bone in EDTA for over a month [15]. Biochemically, it is known that EDTA treatment can remove several non-collagenous organic constituents. Dentin demineralization using the above-mentioned agents not only dissolves the mineral phase in dentin but also induces the hydrolysis of the collagen matrix, which destroys the collagen superstructure. Therefore, the use of EDTA as opposed to an acidic process is preferred for the demineralization of dentin to avoid any damage to the collagen matrix that would be caused by the acidic process [16]. Thus, following treatment with EDTA and guanidine HCl, 2 mm thick completely demineralized dentin blocks that were only composed of collagen matrix. These dentin blocks were employed to evaluate the extent of biomimetic remineralization. This model is different from our previous dentin model that was created by surface acid-etching, which only consisted of a 4–6 μm thick demineralized layer on top of a mineralized dentin substrate [5,6,14,17]. In this case, we also used guanidine HCl to remove NCPs from the collagen matrix [15,16,18]. Thus, the demineralized dentin blocks that were obtained only consisted of collagen matrix.

In the present study, the intertubular and peritubular dentin collagen matrices of the completely demineralized dentin blocks were well remineralized (Figure 4) to form microstructures similar to natural dentin (Figure 2b). However, it was notable that the mineralizing process was quite different between the model of surface demineralized dentin and the model using completely demineralized dentin blocks. In the model that used completely demineralized dentin blocks, a cross-sectional view of the remineralized dentin showed that the dentinal tubules near the surface were deposited with numerous crystals (Figure 4a,c). However, there were virtually no precipitated mineral crystals in the interior of the dentin (Figure 4b,d). Conversely, in the model of surface demineralized dentin, mineralization was found not only in peri-/intertubular dentin collagen fibrils but also in dentinal tubules that were occluded by densely gathered HA crystals [14]. This phenomenon might be a consequence of different remineralization mechanisms. The previous research has indicated that the use of calcium- and phosphate-releasing remineralizing materials may be related to the remineralization of the collagen matrix by the epitaxial growth of calcium phosphate salts on remnant seed crystallites [19]. Epitaxial growth is regarded as a top-down approach according to the classical crystallization theory, which occurs *via* ion-by-ion addition to pre-existing seed crystallites. The partially demineralized model has remnant apatite seed crystallites that can act as templates for mineral deposition, and the orientation of the newly formed mineral lattice is determined by the lattice of the underlying crystal [13]. Thus, the remineralization of a partially demineralized collagen matrix is thermodynamically more favorable than that of a completely demineralized collagen matrix with homogeneous nucleation. HA crystals that formed on the surfaces of tubular walls under the demineralized layer act as seed nuclei to initiate HA crystal formation, which occludes dentinal tubules. This above-mentioned epitaxial growth process also occurred in the remineralization of early enamel carious lesion [2,20]. Contrary to this, there were no HA crystal nuclei in our model of completely demineralized collagen matrix. The generated current accelerates the migration speed of the calcium and phosphate ions and mineralizing precursors in a specific direction. In the completely demineralized dentin slices, the tubules acted as ion channels through which the calcium and phosphate ions could migrate at high speed and flow easily. Thus, the collagen matrix entrap the mineral ions and induce their precipitation. Therefore, more HA crystals were deposited on the dentin collagen surface. The newly formed HA crystals could act as seed nuclei to promote mineralization. Following the infiltration of mineral precursor nanodroplets into the collagen, the precursors solidified with time and could potentially block further precursor infiltration. Finally, the nanodroplets primarily aggregated in the tubules near the dentin surface. Therefore, we found that dentinal tubules near the surface of the dentin slices were covered with numerous deposited crystals, whereas in the interiors of the dentin slices few mineral crystals were found within the tubules (Figure 4b,d).

In biology, a biomineralization process is an organic, matrix particle-mediated, non-classical crystallization pathway [21]. Mineralization using collagen fibrils is often thought of as a bottom-up mineralization approach based on the non-classical theory of crystallization, which involves metastable amorphous mineral precursors and mesocrystals [22,23]. However, the role of the collagen matrix in apatite mineralization remains a matter of debate. Numerous studies paid more attention to the study of NCPs, as they were expected to play an important role in bone and dentin biomineralization [4,24,25,26,27,28]. The results suggested that noncollagenous extracellular matrix proteins are required for the regulation of dentin mineralization and for control of the dimension, order, and hierarchy of apatite deposition within mineralized tissues. Kim *et al.* [23] even reported that the mineralization of completely demineralized dentin did not occur *via* a bottom-up approach when biomimetic analogs were absent from the remineralizing medium. Nevertheless, in the recent years, other voices appeared. For example, Wang Yan *et al.* [29] reported that type I collagen *in vitro* could initiate and orient the growth of carbonated apatite mineral in the absence of any other vertebrate extracellular matrix molecules of calcifying tissues. In addition, the collagen matrix influenced the structural characteristics at the atomic scale and controlled the size and three-dimensional distribution of apatite at larger length scales. These results called into question recent consensus in the literature on the need for Ca-rich non-collagenous proteins for collagen mineralization to occur *in vivo*. Afterwards, Nudelman *et al.* [30] reported that even in the presence of C-DMP1 crystal, nucleation was controlled by collagen itself. In our study, our mineralization system of using agarose hydrogel loaded with calcium phosphate ions could also achieve the intrafibrillar and interfibrillar mineralization without the NCPs in the mineralization medium. However, in the control group (Figure 3e,f), we found that it could not induce dentin collagen mineralization without the aid of an electric field. Based on our previous study [14], we could further prove that the application of a direct electric field can promote the mineralization of collagen fibrils, even in the absence of NCPs or their analogs, for reasons that were discussed earlier [14]. The mechanism behind these findings warrants further study.

## 4. Experimental Section

### 4.1. Dentin Slice Preparation

Human third molars without fillings (extracted following the standard procedures for extraction at the College of Stomatology, Anhui Medical University and handled only after obtaining each patient’s informed consent) were selected. The molars were disinfected with 3% sodium hypochlorite (to remove adhered bacteria) in phosphate-buffered saline. Following this, 2 mm thick dentin slices were prepared by using a low-speed diamond saw (IsoMet Low Speed Saw, Buehler, Lake Bluff, IL, USA) perpendicular to the longitudinal axis of the tooth. The slices were polished with 600-, 1200-, 2400-, and 3000-grit silicon carbide papers and then ultrasonically cleaned with acetone, ethanol, and deionized water. They were stored at 4 °C before treatment.

### 4.2. Preparation of Completely Demineralized Dentin Collagen Matrix Slices

Eleven dentin slices were used in our study. One slice was used to study sound dentin. The remaining ten slices were demineralized in 0.5 M ethylene diamine tetraacetic acid (EDTA) (Acros Organics, Morris Plains, NJ, USA, pH adjusted to 8.0), which contained 0.02% (w/v) sodium azide (Sigma, St. Louis, MO, USA) to avoid bacterial contamination. The dentin slices were incubated in EDTA solution with stirring at room temperature for 20 days. After this treatment, the slices were thoroughly rinsed with deionized water five times for 5 min each and then cleaned ultrasonically for 10 min. The slices were then alternately immersed in 4 M guanidine HCl (Biosharp, Beijing, China, pH 7.4) and 0.05 M Tris-HCl/1 M NaCl (Solarbio, Beijing, China, pH 8.2) for 2 days at 4 °C to remove the NCPs. Following this, the slices were rinsed with deionized water as described above and ultrasonically cleaned for 10 min. One of the demineralized slices was used to study the structure of completely demineralized dentin. Three slices were used as the experimental group and underwent remineralization aided by an electric field for two remineralization cycles. The other three slices were used as the experimental group for six remineralization cycles. The remaining three slices were remineralized without the aid of an electric field and served as the control group.

### 4.3. Preparation of the Mineralizing Medium in Agarose Hydrogel

CaCl_2_-agarose hydrogel was prepared by mixing agarose powder (Regular Agarose G-10, BIOWEST, Nuaille, France) (1.0 g) into 100 mL of a 0.13 M CaCl_2_ solution (CaCl_2_·2H_2_O, Sigma-Aldrich, St. Louis, MO, USA). Na_2_HPO_4_-agarose hydrogel was prepared by mixing agarose powder (1.0 g) into 100 mL of a 0.26 M Na_2_HPO_4_ (Sigma-Aldrich, St. Louis, MO, USA) solution containing 500 ppm fluoride (Sigma-Aldrich, St. Louis, MO, USA). The pH values of the solutions were adjusted to 6.5 using 0.1 M NaOH and 0.1 M HCl. The mixtures were allowed to swell for 30 min and were then heated to 100 °C to completely dissolve the agarose.

### 4.4. Dentin Remineralization Using the Calcium Phosphate Agarose Hydrogel Aided by an Electric Field

The electric field-aided mineralizing system consists of a two-way horizontal polyether tube, two plastic cells, two graphite electrodes, and an electrophoresis power supply (DYY-10C Electrophoresis, Liuyi Instrument Factory, Beijing, China) [14]. The CaCl_2_ hydrogel and the Na_2_HPO_4_ hydrogel were individually placed into opposing sides of the tube and were separated by a demineralized dentin slice. The tube was then connected to the plastic cells. The electrodes were set into the bottom of the cells, which were filled with 0.9% NaCl solution to enhance the electrical conductivity. The electric current was maintained constant at 20 mA during electrophoresis. Both the hydrogels and the NaCl solution were refreshed every 2 h, and the completion of a cycle of mineralization was defined by their exchange. The dentin slice was ultrasonically cleaned for 2 min after each cycle. The sample was taken out after two cycles and again after six cycles for assessment and characterization. For the control group, the above-described mineralizing system was used without the application of an electric field, and the agarose gel was refreshed every other day.

### 4.5. Characterizing the Dentin Slices after Their Remineralization

The composition of the precipitates that formed on the demineralized dentin surface after six cycles of mineralization was evaluated by X-ray diffraction (XRD; X’Pert Pro, Philips Almelo, The Netherlands). A sound dentin slice and a completely demineralized dentin slice were studied for comparison.

The dentin slices after two and six cycles of mineralization were dehydrated using a series of ethanol treatments and were dried in a critical-point evaporator before being sputter-coated with gold for scanning electron microscopy (SEM) analysis. The morphologies and locations of the precipitates were evaluated by field emission scanning electron microscopy (FE-SEM; Sirion 200, FEICo, Hillsboro, OR, USA, or S4800, Hitachi High Technologies America, Inc., Dallas, TX, USA).

The surface of each dentin slice was scratched using a probe after two cycles of mineralization. The collected crumbs were smeared onto a copper grid for analysis by transmission electron microscopy (TEM) (Tecnai G2 20, FEI Co., Hillsboro, OR, USA). Bright-field and selected area electron diffraction (SAED) modes were used without staining the samples prior to analysis.

## 5. Conclusions

A completely demineralized dentin collagen matrix block was used to test an electric field-aided calcium and phosphate agarose hydrogel biomimetic mineralization system. It was further confirmed that the use of an electric field was able to promote the remineralization of dentin collagen matrix. The success of this current proof-of-concept study may provide a new protocol for the treatment of deeper dentinal defects.
